# Hexaaqua­cobalt(II) bis­{[*N*-(4-meth­oxy-2-oxidobenzyl­idene)glycylglycinato]nickel(II)} hexa­hydrate

**DOI:** 10.1107/S1600536809026750

**Published:** 2009-07-18

**Authors:** Jiaxun Jiang, Yao Lu, Limin Yuan, Wenlong Liu

**Affiliations:** a College of Chemistry and Chemical Engineering, Yangzhou Universitry, Yangzhou 225002, People’s Republic of China

## Abstract

In the title compound, [Co(H_2_O)_6_][Ni(C_12_H_11_N_2_O_5_)]_2_·6H_2_O, the Ni^II^ atom has a nearly square-planar coordination with two N and two O atoms of the *N*-(4-meth­oxy-2-oxidobenzyl­idene)glycylglycinate Schiff base ligand (*L* ^3−^). The Co^II^ atom sits on an inversion center and is coordinated to six aqua ligands in a slightly distorted octa­hedral geometry. The [Co(H_2_O)_6_]^2+^ cations and [Ni*L*]^−^ anions form columns along the *a* axis by O—H⋯O hydrogen bonds. Additional hydrogen bonds between the uncoordinated and coordinated water molecules help to consolidate the crystal packing.

## Related literature

For the structures of the copper(II) analogues, see: Liu *et al.* (2006 ▶); Zou *et al.* (2004 ▶). For the magnetic properties of copper(II) heteronuclear complexes, see: Liu *et al.* (2004 ▶); Zou *et al.* (2003 ▶). 
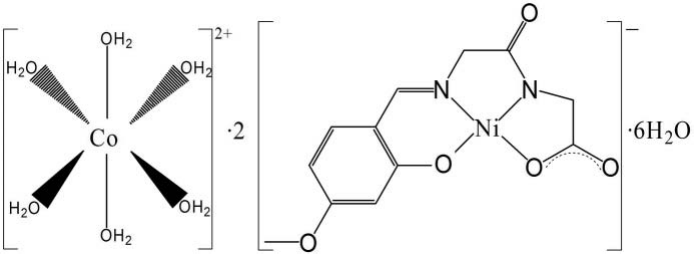


## Experimental

### 

#### Crystal data

[Co(H_2_O)_6_][Ni(C_12_H_11_N_2_O_5_)]_2_·6H_2_O
*M* *_r_* = 919.00
Triclinic, 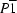

*a* = 7.9052 (8) Å
*b* = 10.7595 (10) Å
*c* = 11.5032 (11) Å
α = 76.325 (1)°
β = 76.654 (1)°
γ = 80.334 (1)°
*V* = 918.34 (15) Å^3^
*Z* = 1
Mo *K*α radiation
μ = 1.55 mm^−1^
*T* = 296 K
0.30 × 0.30 × 0.25 mm

#### Data collection

Bruker SMART APEX CCD diffractometer
Absorption correction: multi-scan (*SADABS*; Sheldrick, 2004 ▶) *T*_min_ = 0.633, *T*_max_ = 0.672
7254 measured reflections
3572 independent reflections
3300 reflections with *I* > 2σ(*I*)
*R*_int_ = 0.073

#### Refinement

*R*[*F* ^2^ > 2σ(*F* ^2^)] = 0.035
*wR*(*F* ^2^) = 0.091
*S* = 1.04
3572 reflections
272 parameters
18 restraints
H atoms treated by a mixture of independent and constrained refinement
Δρ_max_ = 0.67 e Å^−3^
Δρ_min_ = −0.88 e Å^−3^

### 

Data collection: *SMART* (Bruker, 2002 ▶); cell refinement: *SAINT-Plus* (Bruker, 2003 ▶); data reduction: *SAINT-Plus*; program(s) used to solve structure: *SHELXTL* (Sheldrick, 2008 ▶); program(s) used to refine structure: *SHELXTL*; molecular graphics: *SHELXTL*; software used to prepare material for publication: *SHELXTL*.

## Supplementary Material

Crystal structure: contains datablocks I, global. DOI: 10.1107/S1600536809026750/pk2168sup1.cif

Structure factors: contains datablocks I. DOI: 10.1107/S1600536809026750/pk2168Isup2.hkl

Additional supplementary materials:  crystallographic information; 3D view; checkCIF report

## Figures and Tables

**Table 1:** Hydrogen-bond geometry (Å, °)

*D*—H⋯*A*	*D*—H	H⋯*A*	*D*⋯*A*	*D*—H⋯*A*
O6—H6*A*⋯O9	0.831 (16)	1.973 (17)	2.796 (2)	171 (3)
O6—H6*B*⋯O2	0.848 (16)	1.964 (16)	2.809 (2)	175 (3)
O7—H7*A*⋯O3	0.854 (16)	1.890 (17)	2.721 (2)	164 (3)
O7—H7*B*⋯O4^i^	0.844 (16)	2.007 (17)	2.835 (2)	167 (3)
O8—H8*C*⋯O10^ii^	0.827 (16)	1.914 (17)	2.733 (2)	170 (3)
O8—H8*D*⋯O11^ii^	0.844 (16)	1.927 (17)	2.756 (2)	167 (3)
O9—H9*A*⋯O11^iii^	0.850 (17)	2.030 (17)	2.871 (2)	170 (3)
O9—H9*B*⋯O4^iv^	0.824 (17)	2.121 (18)	2.941 (2)	173 (3)
O10—H10*C*⋯O4^v^	0.822 (16)	1.963 (17)	2.775 (2)	169 (3)
O10—H10*D*⋯O3^vi^	0.831 (17)	2.025 (17)	2.840 (2)	167 (3)
O11—H11*B*⋯O1^vii^	0.850 (17)	1.976 (17)	2.817 (2)	170 (3)
O11—H11*A*⋯O10^viii^	0.841 (17)	1.999 (18)	2.778 (2)	154 (3)

Symmetry codes: (i) 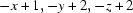; (ii) 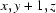; (iii) 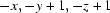; (iv) 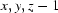; (v) 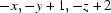; (vi) 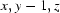; (vii) 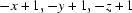; (viii) 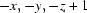.

**Fig. 1.**
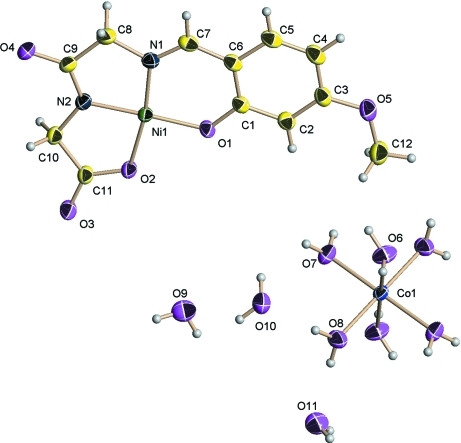
The molecular structure with atom labels and 50% probability displacement ellipsoids. Unlabeled atoms are related to labeled atoms by the symmetry operation (-x + 1, -y + 2, -z + 1).

**Fig. 2.**
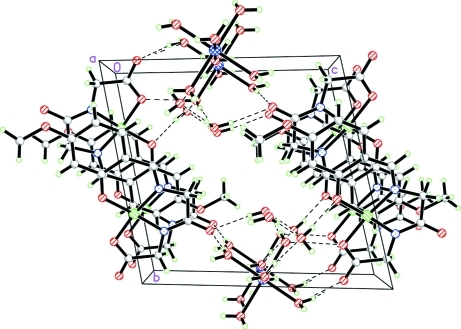
Crystal packing viewed down the a-axis, showing separated stacked columns connected by O—H···O hydrogen bonds (dashed lines).

## supplementary crystallographic information

### Comment

We have reported a series of heteronuclear complexes of Schiff bases synthesized from small peptides. Recently, we reported the copper(II) heteronuclear complexes of Schiff base ligands resulting from the condensation of simple peptides with salicylaldehyde (Liu *et al.*, 2006; Zou *et al., *2004) and magnetic properties (Liu *et al.*, 2004; Zou *et al.*, 2003). Hydrogen bonds play an important role in these complexes. Now we report the synthesis and structure of Ni(II)—Co(II) Schiff base complex derived from glycylglycine and 4-methoxy-salicylaldehyde. The heteronuclear complex crystallizes in the triclinic space group *P*1. The asymmetric unit consists of one [NiL]^-^ anion (*L* is a Schiff base derived from glycylglycine and 4-methoxy-salicylaldehyde), half a Co(H_2_O)_6_^2+^ cation [Co(1), O(6), O(7), O(8)] and three uncoordinated water molecules [O(9), O(10), O(11)] (Fig. 1). [NiL]^- ^ has an approximate square-planar geometry. The deprotonated Schiff base is a triple negatively charged tetradentate ONNO ligand, coordinating to the Ni(II) center by one phenolate O atom [O(1)], one imine N atom [N(1)], one deprotonated amide N atom [N(2)] and one carboxylate O atom [O(3)]. The benzene ring [C(1)–C(6)] and the O(1), C(1), C(6), C(7), N(1), Ni(1) chelate ring are almost coplanar with a dihedral angle of 0.55 (7)°, suggesting a large π-electron delocalization. The Cobalt(II) atom lies on an inversion center and the coordination by six aqua ligands is slightly distorted octahedral. The six Co—O bonds in the structure are in the range of 2.0518 (13) - 2.0711 (14) Å. O—H···O hydrogen bonds (Table 1) play an important role in the stabilization of the crystal structure. The anions and cations form hydrogen bonded columns along the *a*-axis (Fig. 2). These are well segregated from each other.

### Experimental

Glycylglycine (5 mmol), 4-methoxy-salicylaldehyde (5 mmol) and LiOH (10 mmol) were dissolved in MeOH/H_2_O (30 ml, v:v = 1:1) and refluxed for 30 min. Then Ni(ClO_4_)_2_.6H_2_O (5 mmol) was added to the solution and the resulting solution was adjusted to pH 9–11 by 5 mol/*L* NaOH solution. After stirring at room temperature for 1 h, CoCl_2_.6H_2_O (2.5 mmol) was added. A yellow precipitate formed immediately, and was stirred for another 30 min and then filtere. The precipitate was recrystallized from water. Yellow crystals suitable for X-ray diffraction were obtained after one week. (yield 30% based on Ni(ClO_4_)_2_.6H_2_O).

### Refinement

The water H atoms were located in a difference Fourier map with a distance restraint of O—H = 0.85 Å and *U*_iso_(H) =1.5*U*_eq_(O). The refinement of water H atoms were performed using 18 least-squares restraints by applying *DFIX* instructions of *SHELXTL*. All other H atoms were positioned geometrically and constrained as riding atoms, with C—H distances of 0.93–0.97 Å and *U*_iso_(H) set to 1.2 or 1.5_eq_(C) of the parent atom.

### Crystal data

**Table:**

[Co(H_2_O)_6_][Ni(C_12_H_11_N_2_O_5_)]_2_·6H_2_O	*Z* = 1
*M**_r_* = 919.00	*F*(000) = 477
Triclinic, *P*1	*D*_x_ = 1.662 Mg m^−^^3^
Hall symbol: -P 1	Mo *K*α radiation, λ = 0.71073 Å
*a* = 7.9052 (8) Å	Cell parameters from 6194 reflections
*b* = 10.7595 (10) Å	θ = 2.4–28.4°
*c* = 11.5032 (11) Å	µ = 1.55 mm^−^^1^
α = 76.325 (1)°	*T* = 296 K
β = 76.654 (1)°	Block, yellow
γ = 80.334 (1)°	0.30 × 0.30 × 0.25 mm
*V* = 918.34 (15) Å^3^	

### Data collection

**Table:**

Bruker SMART APEX CCD diffractometer	3572 independent reflections
Radiation source: sealed tube	3300 reflections with *I* > 2σ(*I*)
graphite	*R*_int_ = 0.073
φ and ω scans	θ_max_ = 26.0°, θ_min_ = 1.9°
Absorption correction: multi-scan (*SADABS*; Sheldrick, 2004)	*h* = −9→9
*T*_min_ = 0.633, *T*_max_ = 0.672	*k* = −13→13
7254 measured reflections	*l* = −14→14

### Refinement

**Table:**

Refinement on *F*^2^	Primary atom site location: structure-invariant direct methods
Least-squares matrix: full	Secondary atom site location: difference Fourier map
*R*[*F*^2^ > 2σ(*F*^2^)] = 0.035	Hydrogen site location: inferred from neighbouring sites
*wR*(*F*^2^) = 0.091	H atoms treated by a mixture of independent and constrained refinement
*S* = 1.04	*w* = 1/[σ^2^(*F*_o_^2^) + (0.0608*P*)^2^ + 0.0666*P*] where *P* = (*F*_o_^2^ + 2*F*_c_^2^)/3
3572 reflections	(Δ/σ)_max_ = 0.002
272 parameters	Δρ_max_ = 0.67 e Å^−^^3^
18 restraints	Δρ_min_ = −0.88 e Å^−^^3^

### Special details

**Table:**

Geometry. All e.s.d.'s (except the e.s.d. in the dihedral angle between two l.s. planes) are estimated using the full covariance matrix. The cell e.s.d.'s are taken into account individually in the estimation of e.s.d.'s in distances, angles and torsion angles; correlations between e.s.d.'s in cell parameters are only used when they are defined by crystal symmetry. An approximate (isotropic) treatment of cell e.s.d.'s is used for estimating e.s.d.'s involving l.s. planes.
Refinement. Refinement of *F*^2^ against ALL reflections. The weighted *R*-factor *wR* and goodness of fit *S* are based on *F*^2^, conventional *R*-factors *R* are based on *F*, with *F* set to zero for negative *F*^2^. The threshold expression of *F*^2^ > σ(*F*^2^) is used only for calculating *R*-factors(gt) *etc*. and is not relevant to the choice of reflections for refinement. *R*-factors based on *F*^2^ are statistically about twice as large as those based on *F*, and *R*- factors based on ALL data will be even larger.

### Fractional atomic coordinates and isotropic or equivalent isotropic displacement parameters (Å^2^)

**Table:**

	*x*	*y*	*z*	*U*_iso_*/*U*_eq_	
Ni1	0.53859 (3)	0.70463 (2)	0.993860 (18)	0.02321 (10)	
Co1	0.5000	1.0000	0.5000	0.02262 (11)	
C1	0.7940 (2)	0.51644 (17)	0.89989 (17)	0.0253 (4)	
C2	0.9034 (2)	0.46971 (18)	0.79967 (18)	0.0292 (4)	
H2	0.9018	0.5164	0.7207	0.035*	
C3	1.0127 (2)	0.35529 (19)	0.81779 (19)	0.0302 (4)	
C4	1.0201 (3)	0.28302 (19)	0.9361 (2)	0.0349 (4)	
H4	1.0962	0.2073	0.9477	0.042*	
C5	0.9134 (3)	0.32634 (18)	1.03332 (19)	0.0329 (4)	
H5	0.9168	0.2780	1.1115	0.039*	
C6	0.7977 (2)	0.44218 (17)	1.01975 (17)	0.0273 (4)	
C7	0.6938 (2)	0.48078 (18)	1.12730 (18)	0.0295 (4)	
H7	0.7051	0.4269	1.2020	0.035*	
C8	0.4866 (2)	0.6148 (2)	1.24644 (17)	0.0337 (4)	
H8A	0.5669	0.6223	1.2960	0.040*	
H8B	0.4162	0.5464	1.2908	0.040*	
C9	0.3700 (2)	0.74026 (19)	1.22086 (16)	0.0268 (4)	
C10	0.2913 (2)	0.90964 (18)	1.05217 (17)	0.0290 (4)	
H10A	0.3237	0.9817	1.0760	0.035*	
H10B	0.1664	0.9068	1.0811	0.035*	
C11	0.3377 (2)	0.92460 (18)	0.91472 (17)	0.0282 (4)	
C12	1.1187 (3)	0.3666 (2)	0.6024 (2)	0.0459 (5)	
H12A	1.0029	0.3720	0.5875	0.069*	
H12B	1.2000	0.3187	0.5483	0.069*	
H12C	1.1511	0.4518	0.5884	0.069*	
N1	0.58553 (19)	0.58460 (15)	1.12880 (14)	0.0268 (3)	
N2	0.3876 (2)	0.79006 (16)	1.10386 (14)	0.0273 (3)	
O1	0.69326 (16)	0.62706 (13)	0.87706 (12)	0.028	
O2	0.45819 (18)	0.84079 (13)	0.87239 (11)	0.0307 (3)	
O3	0.26077 (19)	1.01400 (14)	0.84986 (12)	0.0393 (3)	
O4	0.27210 (18)	0.78848 (15)	1.30738 (12)	0.0342 (3)	
O5	1.12210 (19)	0.30305 (15)	0.72600 (14)	0.0408 (3)	
O6	0.4645 (2)	0.83132 (15)	0.62941 (13)	0.0392 (3)	
H6A	0.387 (3)	0.794 (3)	0.618 (2)	0.059*	
H6B	0.457 (4)	0.831 (3)	0.7041 (17)	0.059*	
O7	0.46103 (19)	1.10891 (15)	0.63028 (12)	0.0339 (3)	
H7A	0.386 (3)	1.093 (3)	0.6968 (19)	0.051*	
H7B	0.551 (2)	1.129 (3)	0.645 (2)	0.051*	
O8	0.23876 (17)	1.02599 (15)	0.49286 (13)	0.0361 (3)	
H8C	0.162 (3)	1.018 (3)	0.5560 (17)	0.054*	
H8D	0.208 (3)	1.089 (2)	0.4394 (19)	0.054*	
O9	0.2328 (2)	0.69532 (17)	0.57292 (15)	0.0458 (4)	
H9A	0.127 (3)	0.722 (3)	0.601 (2)	0.069*	
H9B	0.251 (4)	0.717 (3)	0.4978 (15)	0.069*	
O10	0.00613 (19)	0.01756 (16)	0.71029 (14)	0.0415 (4)	
H10C	−0.067 (3)	0.081 (2)	0.699 (3)	0.062*	
H10D	0.073 (3)	0.029 (3)	0.752 (3)	0.062*	
O11	0.1080 (2)	0.20515 (16)	0.31073 (15)	0.0446 (4)	
H11B	0.178 (3)	0.248 (3)	0.254 (2)	0.067*	
H11A	0.080 (4)	0.150 (2)	0.280 (3)	0.067*	

### Atomic displacement parameters (Å^2^)

**Table:**

	*U*^11^	*U*^22^	*U*^33^	*U*^12^	*U*^13^	*U*^23^
Ni1	0.02687 (14)	0.02163 (15)	0.01832 (14)	0.00209 (10)	−0.00428 (10)	−0.00239 (10)
Co1	0.02615 (18)	0.0238 (2)	0.01660 (18)	−0.00234 (14)	−0.00260 (13)	−0.00383 (13)
C1	0.0255 (8)	0.0193 (8)	0.0313 (9)	−0.0020 (6)	−0.0082 (7)	−0.0040 (7)
C2	0.0315 (9)	0.0232 (9)	0.0317 (10)	−0.0001 (7)	−0.0080 (7)	−0.0042 (7)
C3	0.0282 (8)	0.0240 (9)	0.0398 (11)	−0.0004 (7)	−0.0075 (8)	−0.0108 (8)
C4	0.0354 (9)	0.0230 (9)	0.0459 (12)	0.0035 (7)	−0.0143 (9)	−0.0048 (8)
C5	0.0369 (9)	0.0228 (10)	0.0387 (10)	−0.0010 (8)	−0.0158 (8)	0.0002 (8)
C6	0.0285 (8)	0.0198 (9)	0.0336 (10)	−0.0019 (7)	−0.0106 (7)	−0.0019 (7)
C7	0.0316 (8)	0.0253 (9)	0.0292 (9)	−0.0038 (7)	−0.0102 (7)	0.0036 (7)
C8	0.0333 (9)	0.0425 (11)	0.0222 (9)	−0.0015 (8)	−0.0066 (7)	−0.0020 (8)
C9	0.0269 (8)	0.0333 (10)	0.0215 (8)	−0.0059 (7)	−0.0044 (7)	−0.0067 (7)
C10	0.0351 (9)	0.0254 (9)	0.0244 (9)	0.0011 (7)	−0.0031 (7)	−0.0067 (7)
C11	0.0328 (9)	0.0238 (9)	0.0249 (9)	0.0013 (7)	−0.0026 (7)	−0.0053 (7)
C12	0.0519 (12)	0.0402 (13)	0.0409 (12)	0.0061 (10)	−0.0034 (10)	−0.0130 (10)
N1	0.0278 (7)	0.0279 (8)	0.0227 (7)	−0.0017 (6)	−0.0065 (6)	−0.0012 (6)
N2	0.0324 (7)	0.0260 (8)	0.0220 (7)	0.0015 (6)	−0.0053 (6)	−0.0053 (6)
O1	0.031	0.023	0.024	0.006	−0.004	−0.001
O2	0.0386 (7)	0.0272 (7)	0.0203 (6)	0.0076 (5)	−0.0038 (5)	−0.0035 (5)
O3	0.0491 (8)	0.0322 (8)	0.0258 (7)	0.0151 (6)	−0.0042 (6)	−0.0019 (6)
O4	0.0367 (7)	0.0427 (8)	0.0218 (7)	−0.0003 (6)	−0.0036 (5)	−0.0091 (6)
O5	0.0438 (8)	0.0324 (8)	0.0418 (8)	0.0109 (6)	−0.0068 (7)	−0.0116 (7)
O6	0.0556 (9)	0.0372 (8)	0.0253 (7)	−0.0144 (7)	−0.0108 (6)	0.0014 (6)
O7	0.0368 (7)	0.0401 (8)	0.0258 (7)	−0.0076 (6)	0.0001 (5)	−0.0129 (6)
O8	0.0303 (7)	0.0435 (9)	0.0288 (7)	0.0000 (6)	−0.0052 (5)	−0.0005 (6)
O9	0.0518 (9)	0.0448 (10)	0.0398 (9)	−0.0103 (8)	−0.0107 (7)	−0.0022 (7)
O10	0.0363 (7)	0.0485 (10)	0.0365 (8)	0.0039 (7)	−0.0028 (6)	−0.0128 (7)
O11	0.0486 (9)	0.0420 (9)	0.0361 (8)	−0.0067 (7)	−0.0053 (7)	0.0037 (7)

### Geometric parameters (Å, °)

**Table:**

Ni1—N2	1.8318 (15)	C8—H8A	0.9700
Ni1—N1	1.8364 (15)	C8—H8B	0.9700
Ni1—O1	1.8510 (13)	C9—O4	1.270 (2)
Ni1—O2	1.9058 (13)	C9—N2	1.310 (2)
Co1—O7^i^	2.0518 (13)	C10—N2	1.451 (2)
Co1—O7	2.0518 (13)	C10—C11	1.514 (3)
Co1—O8	2.0551 (13)	C10—H10A	0.9700
Co1—O8^i^	2.0551 (13)	C10—H10B	0.9700
Co1—O6	2.0711 (14)	C11—O3	1.235 (2)
Co1—O6^i^	2.0711 (14)	C11—O2	1.289 (2)
C1—O1	1.323 (2)	C12—O5	1.429 (3)
C1—C2	1.413 (3)	C12—H12A	0.9600
C1—C6	1.425 (3)	C12—H12B	0.9600
C2—C3	1.382 (3)	C12—H12C	0.9600
C2—H2	0.9300	O6—H6A	0.831 (16)
C3—O5	1.369 (2)	O6—H6B	0.848 (16)
C3—C4	1.408 (3)	O7—H7A	0.854 (16)
C4—C5	1.363 (3)	O7—H7B	0.844 (16)
C4—H4	0.9300	O8—H8C	0.827 (16)
C5—C6	1.416 (3)	O8—H8D	0.844 (16)
C5—H5	0.9300	O9—H9A	0.850 (17)
C6—C7	1.427 (3)	O9—H9B	0.824 (17)
C7—N1	1.289 (2)	O10—H10C	0.822 (16)
C7—H7	0.9300	O10—H10D	0.831 (17)
C8—N1	1.477 (2)	O11—H11B	0.850 (17)
C8—C9	1.509 (3)	O11—H11A	0.841 (17)
			
N2—Ni1—N1	85.19 (7)	C9—C8—H8A	110.0
N2—Ni1—O1	176.83 (6)	N1—C8—H8B	110.0
N1—Ni1—O1	97.51 (6)	C9—C8—H8B	110.0
N2—Ni1—O2	85.41 (6)	H8A—C8—H8B	108.4
N1—Ni1—O2	170.42 (6)	O4—C9—N2	126.66 (18)
O1—Ni1—O2	91.94 (5)	O4—C9—C8	121.02 (16)
O7^i^—Co1—O7	180.000 (1)	N2—C9—C8	112.30 (16)
O7^i^—Co1—O8	87.11 (6)	N2—C10—C11	107.22 (14)
O7—Co1—O8	92.89 (6)	N2—C10—H10A	110.3
O7^i^—Co1—O8^i^	92.89 (6)	C11—C10—H10A	110.3
O7—Co1—O8^i^	87.11 (6)	N2—C10—H10B	110.3
O8—Co1—O8^i^	180.000 (1)	C11—C10—H10B	110.3
O7^i^—Co1—O6	87.25 (6)	H10A—C10—H10B	108.5
O7—Co1—O6	92.75 (6)	O3—C11—O2	123.75 (17)
O8—Co1—O6	90.07 (6)	O3—C11—C10	119.65 (16)
O8^i^—Co1—O6	89.93 (6)	O2—C11—C10	116.61 (15)
O7^i^—Co1—O6^i^	92.75 (6)	O5—C12—H12A	109.5
O7—Co1—O6^i^	87.25 (6)	O5—C12—H12B	109.5
O8—Co1—O6^i^	89.93 (6)	H12A—C12—H12B	109.5
O8^i^—Co1—O6^i^	90.07 (6)	O5—C12—H12C	109.5
O6—Co1—O6^i^	180.00 (6)	H12A—C12—H12C	109.5
O1—C1—C2	118.00 (16)	H12B—C12—H12C	109.5
O1—C1—C6	123.54 (17)	C7—N1—C8	119.95 (16)
C2—C1—C6	118.46 (17)	C7—N1—Ni1	125.62 (13)
C3—C2—C1	120.68 (18)	C8—N1—Ni1	114.43 (12)
C3—C2—H2	119.7	C9—N2—C10	124.56 (16)
C1—C2—H2	119.7	C9—N2—Ni1	119.54 (13)
O5—C3—C2	124.42 (18)	C10—N2—Ni1	115.88 (12)
O5—C3—C4	114.44 (17)	C1—O1—Ni1	125.20 (11)
C2—C3—C4	121.15 (18)	C11—O2—Ni1	114.57 (11)
C5—C4—C3	118.64 (18)	C3—O5—C12	118.60 (16)
C5—C4—H4	120.7	Co1—O6—H6A	111 (2)
C3—C4—H4	120.7	Co1—O6—H6B	120 (2)
C4—C5—C6	122.47 (18)	H6A—O6—H6B	113 (2)
C4—C5—H5	118.8	Co1—O7—H7A	121.4 (18)
C6—C5—H5	118.8	Co1—O7—H7B	117.0 (18)
C5—C6—C1	118.58 (17)	H7A—O7—H7B	109 (2)
C5—C6—C7	118.28 (17)	Co1—O8—H8C	121.2 (18)
C1—C6—C7	123.11 (17)	Co1—O8—H8D	115.3 (19)
N1—C7—C6	124.99 (17)	H8C—O8—H8D	111 (2)
N1—C7—H7	117.5	H9A—O9—H9B	109 (2)
C6—C7—H7	117.5	H10C—O10—H10D	111 (2)
N1—C8—C9	108.53 (15)	H11B—O11—H11A	107 (2)
N1—C8—H8A	110.0		

Symmetry codes: (i) −*x*+1, −*y*+2, −*z*+1.

### Hydrogen-bond geometry (Å, °)

**Table:**

*D*—H···*A*	*D*—H	H···*A*	*D*···*A*	*D*—H···*A*
O6—H6A···O9	0.83 (2)	1.97 (2)	2.796 (2)	171 (3)
O6—H6B···O2	0.85 (2)	1.96 (2)	2.809 (2)	175 (3)
O7—H7A···O3	0.85 (2)	1.89 (2)	2.721 (2)	164 (3)
O7—H7B···O4^ii^	0.84 (2)	2.01 (2)	2.835 (2)	167 (3)
O8—H8C···O10^iii^	0.83 (2)	1.91 (2)	2.733 (2)	170 (3)
O8—H8D···O11^iii^	0.84 (2)	1.93 (2)	2.756 (2)	167 (3)
O9—H9A···O11^iv^	0.85 (2)	2.03 (2)	2.871 (2)	170 (3)
O9—H9B···O4^v^	0.82 (2)	2.12 (2)	2.941 (2)	173 (3)
O10—H10C···O4^vi^	0.82 (2)	1.96 (2)	2.775 (2)	169 (3)
O10—H10D···O3^vii^	0.83 (2)	2.03 (2)	2.840 (2)	167 (3)
O11—H11B···O1^viii^	0.85 (2)	1.98 (2)	2.817 (2)	170 (3)
O11—H11A···O10^ix^	0.84 (2)	2.00 (2)	2.778 (2)	154 (3)

Symmetry codes: (ii) −*x*+1, −*y*+2, −*z*+2; (iii) *x*, *y*+1, *z*; (iv) −*x*, −*y*+1, −*z*+1; (v) *x*, *y*, *z*−1; (vi) −*x*, −*y*+1, −*z*+2; (vii) *x*, *y*−1, *z*; (viii) −*x*+1, −*y*+1, −*z*+1; (ix) −*x*, −*y*, −*z*+1.
